# The role of external actors in shaping migrant health insurance in Thailand

**DOI:** 10.1371/journal.pone.0234642

**Published:** 2020-07-02

**Authors:** Chantal Herberholz

**Affiliations:** Centre for Health Economics, Faculty of Economics, Chulalongkorn University, Bangkok, Thailand; Tulane University, UNITED STATES

## Abstract

The role of external actors in national health policy in aid-independent countries has received relatively little attention in the literature, despite the fact that influence continues to be exerted once financial support is curtailed as countries graduate from lower income status. Focusing on a specific health policy in an aid-independent country, this qualitative study explores the role of external actors in shaping Thailand’s migrant health insurance. Primary data were collected through in-depth interviews with eighteen key informants from September 2018 to January 2019. The data were analysed using thematic analysis, focusing on three channels of influence, financial resources, technical expertise and inter-sectoral leverage, and their effect on the different stages of the policy process. Given Thailand’s export orientation and the importance of reputational effects, inter-sectoral leverage, mainly through the US TIP Reports and the EU carding decision, emerged as a very powerful channel of influence on priority setting, as it indirectly affected the migrant health insurance through efforts aimed at dealing with problems of human trafficking in the context of labour migration, especially after the 2014 coup d'état. This study helps understand the changed role external actors can play in filling health system gaps in aid-independent countries.

## 1. Introduction

Analyses of international influences on country health priorities and policies mostly focus on developing countries that receive substantive external funding and/or emphasize financial resources as a channel of influence [1–7]. The more dependent a recipient country is on external funding, the larger the positive or negative influence of donors typically is. Financial power arises from the availability and control of financial resources and the resulting ability to determine their allocation [3, 8, 9].

Over the past two decades more and more countries graduated from low-income to middle-income status. Middle-income countries are better able to mobilize domestic resources and, therefore, become less dependent on development aid. International influence, however, prevails once financial support from donors is waning [10, 11]. Apart from financial resources, technical expertise and inter-sectoral leverage are additional channels through which the various stages of the policy process have been influenced [3, 12]. Technical expertise, which encapsulates control over and coordination of knowledge and information generation and use, gives rise to epistemic power [13, 14]. Inter-sectoral leverage, on the other hand, originates from power to influence sectors other than the health sector, such as, for example, the power to impose travel and trade restrictions or to cause reputational damages in response to a country’s failure to address donor priorities [3].

This study explains how external actors had an effect on the different stages of the health policy process in an aid-independent middle-income country, focusing on the case of Thailand’s migrant health insurance and reforms thereof. A better understanding of the channels of influence in aid-independent countries is important for domestic and external actors to promote health policies that fill existing gaps.

Financial resources contributed by donors are rather negligible in Thailand, making up only 0.1 to 0.3 percent of total health expenditures between 1994 and 2011 [15]. The bulk of this came from The Global Fund to Fight AIDS, Tuberculosis and Malaria (GFATM) for HIV/AIDS interventions, especially for migrant workers. Of an estimated 4.9 million non-Thai population in 2018, 3.9 million were migrant workers from the CLMV countries, Cambodia, the Lao People’s Democratic Republic (PDR), Myanmar and Viet Nam, comprising documented (79%) and undocumented (21%) migrant workers [16]. Documented (or registered) migrant workers are regular migrant workers, who registered with the Ministry of Interior (MoI) and hold a work permit issued by the Ministry of Labour (MoL), and migrant workers who came to Thailand under bilateral Memoranda of Understanding (MoU) with CLMV countries [17, 18]. Included are also migrant workers who entered the country irregularly, but then completed the registration process during amnesty periods. Undocumented (or unregistered) migrant workers are irregular migrant workers, who work in Thailand without valid visa and work permit and failed to register during amnesty periods [18].

Thailand is one of the few developing countries that has offered a migrant health insurance scheme to migrants irrespective of their migration status. With the introduction of the tax-based Universal Health Coverage Scheme (UCS) in 2001, Thailand achieved universal health coverage by covering Thai citizens who were neither protected by the Civil Servant Medical Benefit Scheme for civil servants and their dependants nor the Social Security Scheme (SSS) for private sector employees. In the same year, the government started to take a more liberal stance towards the registration of irregular migrant workers and an additional cabinet resolution for the migrant health insurance, that was first introduced in some areas in 1998, was passed, requiring all registered migrant workers to undergo health screening and to purchase the migrant health insurance of the Ministry of Public Health (MHI) [19, 20]. The registration system for migrant workers was revised in 2004 [19], triggering a breakthrough, and registered migrant workers should either be covered by the contributory SSS, which is financed by payroll taxes, or the MHI in case of SSS exemption. The MHI is a prepayment scheme, financed through annual premiums paid by migrants as shown in Table 1, with a comprehensive benefit package [21].

**Table 1:** Migrant health insurance premiums since 2001.

Year	Health check-up		Annual premium	
	THB	USD	THB	USD
Migrant health insurance of the MoPH				
2001 (registered migrant workers)	300	9	1,200	38
2004 (registered migrant workers)	600	19	1,300	41
2013 (migrants)	600	19	2,200	69
2014 (OSSC registered migrants)	500	16	1,600	50
Migrant health insurance of the MoPH for children of migrants				
2013			365	11
32THB/USD				

[19, 20, 22, 23]

Following a 2013 cabinet resolution, unregistered migrants and their dependants could opt in on a voluntary basis, subject to approval by the contracted hospital selling the MHI [20, 23]. In the same year, the benefit package was expanded to also include antiretroviral treatment (ART) for people living with HIV/AIDS [20] and a separate health insurance for migrant children below the age of seven was introduced [23]. Nevertheless, almost 50 percent of migrants from CLMV, with migrants from CLM initially constituting the target beneficiaries of the MHI, were not enrolled in either scheme [24]. Given the involvement of several ministries, a multi-sectoral approach was eventually adopted in 2014, following an order of the National Council for Peace and Order (NCPO, the military-led government that was set up after the coup d'état in May 2014) to expedite administrative processes. Migrant workers from CLM and their dependants, who registered through so-called One Stop Service Centres (OSSC), were required to purchase the MHI as part of the registration process [20, 23].

The policy changes that have taken place since 2001, when the UCS was launched, are the result of internal and external forces that pushed the government to provide better healthcare for migrants [21, 23]. Internally, policies on migrant healthcare have been strongly influenced by cabinet resolutions on migrant worker registration, related laws and regulations, which have implications for health insurance entitlement [20, 21]. According to Suphanchaimat et al. [23], migrant policies and migrant health insurance policies were primarily driven by (i) public health concerns, (ii) national security concerns and (iii) economic necessity as Thailand’s economy is dependent on unskilled labour from neighbouring countries. Allegations of human trafficking by international organizations were identified as an important external factor [21, 23, 25]. Yet, these external forces are more complex and it is unclear through which channels they work and how they affect the different stages of the policy process. As an export-dependent, open economy, external influence is likely to be primarily exerted through inter-sectoral leverage. In addition, the 2014 coup d'état made Thailand the only country in the world ruled by a “*coup-installed military government*” [26], seriously challenging the country’s international reputation. Post-coup reactions may, therefore, have intensified outside pressure.

## 2. Materials and methods

Data were collected through a review of relevant published and grey literature, as well as key informant interviews. Eighteen key informant interviews were conducted in Thailand from September 2018 to January 2019. Initially, ten key informants (primary key informants) were identified purposively, based on their influence on and familiarity with the policy process underpinning the MHI in Thailand, through web-searching and a review of policy and research documents. Included in the initial list are high-level key informants from non-governmental organizations (NGOs), academia, international organizations and government to capture multiple perspectives. Of the ten primary key informants, seven agreed to participate in this study. Further key informants were subsequently identified using the snowball technique. In total, eleven secondary key informants took part in this study. In-depth interviews were conducted until data saturation was reached. Characteristics of key informants are shown in Table 2. To ensure confidentiality, neither names nor affiliation details of individuals are disclosed.

**Table 2:** Key informants.

Code	Affiliation	Role	Total	Male	Female
KI 8, 9, 12, 13, 16	Government ministry (MoPH, MoL)	Decision-maker	5	4	1
KI 4	Semi-autonomous agency	Researcher	1	1	0
KI 1, 6, 7, 10	University	Researcher	4	3	1
KI 2, 3, 14, 15, 18	International organization	Senior officer	5	1	4
KI 5	Domestic NGO	Board member	1	1	0
KI 11, 17	International NGO	Founder	2	2	0
			18	12	6

The interview guide (S1 File), which was developed for the data collection, contains questions about (i) the respondent’s role and how it relates to health insurance for migrants in Thailand, (ii) health insurance for migrants in Thailand, and (iii) external influences on health insurance for migrants in Thailand in general and the MHI in particular.

Interviews were conducted by the researcher in the English language to avoid translation problems and allow a straightforward analysis of the verbatim transcriptions and the field notes. One interview, however, was conducted in the presence of a translator, who translated questions and responses from English to Thai and vice versa on the spot to avoid any miscommunication. Sixteen interviews were conducted in person, mostly at the workplace of the key informants or the workplace of the researcher, one over the telephone and one using a videoconferencing service. Each interview took about one hour to complete.

Ethical approval was obtained from the Ethics Review Committee Involving Human Research Subjects, Health Science Group, Chulalongkorn University on September 7, 2018 (certificate of approval number 208/2018). The requirement to obtain a signed informed consent sheet from participants was waived by the ethics review committee as this research project presents no more than minimal risk to study participants. Verbal consent was obtained from all key informants prior to the interviews, that is after reading out loud the participant information sheet and answering any questions key informants may have had. Verbal consent was documented by the researcher on a separate verbal consent documentation page and subsequently de-identified. Prior to each interview, verbal consent was also obtained to record the interview.

The data were examined using a hybrid approach of deductive and inductive thematic analysis. External actors were defined very broadly to capture all transnational influences, including for example the influence of foreign governments, international organizations, global health initiatives etc. Power is an important concept in health policy, although the notion of power and, hence, the means by which it is exercised have remained contested [8]. Among the influential approaches to understanding power are the three faces of power, that is power as decision-making, power as agenda-setting and power as preference-shaping [9, 27, 28]. The first face of power is based on Dahl’s work [28] and focuses on the overt exercise of power to influence decision-making on specific issues. Bachrach and Baratz [27] added a second face of power to Dahl’s work [28] by considering the absence of issues from political agendas, that is non-decision-making, and the power through agenda-setting. The third face of power, suggested by Lukes [9], emphasizes the power of ideas, beliefs and values to influence preferences and wants of others.

Power relations are based on power resources such as, for instance, financial resources, knowledge, framing and rhetoric, which are important in power analysis [29–33]. Framing refers to how an issue is publicly portrayed by actors to attract attention, shape understanding of an issue and support solutions [34–37]. Several studies highlight the importance of issue framing for health agenda setting, as well as the alignment of issues with favourable norms through framing [36, 38–44].

Nye [45] distinguishes between hard power in international relations such as force, payment and agenda-setting based on these, and soft power like persuasion and attraction. Each of the three faces of power allows hard and soft methods, ranging from coercion to attraction [45, 46]. Trade-related measures such as trade embargoes and withdrawal of trade preferences are examples of harder power that have been employed by countries to coerce non-complying countries to adhere to their values and to international norms [47]. Naming (public identification) and shaming (public condemnation) approaches, on the other hand, are examples of softer power used to externalize non-economic values, including human rights [48, 49]. The three instruments of naming, shaming and sanctioning have been used simultaneously or sequentially to pressure countries to change their behaviour [50]. Kelley’s [51] concept of scorecard diplomacy, an example of which are the US Trafficking in Persons (TIP) reports, goes further by not only naming and shaming non-complying countries through scoring, but also starting a dialogue and giving assistance to improve the score in the future.

Following Khan et al. [3], whose work is largely based on the conceptualization of power by Lukes [9], content was initially grouped according to three channels of influence, that is financial resources, technical expertise and inter-sectoral leverage, and the policy process, which according to the stages heuristic model [52] comprises the four stages of priority setting, policy formulation, policy implementation and monitoring and evaluation. While technical expertise manifests itself as a channel of influence through knowledge and information generation, use and dissemination, inter-sectoral leverage works through sectors other than the health sector such as, for example, through reputational effects that impact international trade [3, 8, 13, 14].

## 3. Results

Thailand is an active member of the regional and global health community [53]. Success stories such as its UCS have been showcased regionally and globally. However, while universal health coverage has been achieved for Thai citizens, migrants and stateless people are not covered by the UCS. Of the approximately 3.9 million migrants from CLMV countries in Thailand, 862,870 were enrolled in the MHI and 1,107,426 in the SSS, implying that about 36 percent of documented (about 50 percent of documented and undocumented) CLMV migrants did not have health insurance in 2018 [24].

Covering migrants is, therefore, one of the final steps to ensure that no one is left behind without health insurance coverage. Migrants who do not have any health insurance have nevertheless received care at public healthcare providers for humanitarian reasons and to prevent the spread of communicable diseases. Migrants are typically asked to pay whatever they can pay. Thus, the burden of any uncovered costs falls on public healthcare providers, most of which are under the Ministry of Public Health (MoPH) [KI 3, KI 5, KI 6, KI 7, KI 8, KI 15, KI 16]. Rising concerns about the financing of charity care became increasingly apparent at the end of the 1990s.

Financial as well as public health concerns of the MoPH were identified by key informants as the main factors that influenced the MHI during its early stages, together with the importance of unskilled migrant workers from neighbouring countries for the Thai economy in general since the 1990s as discussed in, for example, Suphanchaimat et al. [23]. Notwithstanding, all key informants recognized that external actors also played some role, especially after 2013, and their influences will be discussed in the following part, which is structured according to the channels of influence, that is financial resources, technical expertise and inter-sectoral leverage, as well as the policy process.

### 3.1 Financial resources

Although external health expenditure has been of minor importance in Thailand, some key informants explicitly acknowledged the role of the GFATM. The GFATM inter alia provided financial support for HIV/AIDS prevention interventions and treatment, focusing on migrant workers through its two Prevention of HIV/AIDS among Migrant Workers in Thailand projects (PHAMIT I and II) from 2004 to 2014 [54–56]. Both GFATM-funded projects, which were implemented through strong networks of local and international NGOs, were perceived crucial drivers for the inclusion of ART into the MHI, as well as the expansion of the MHI to ultimately include all migrants, irrespective of their migration status [KI 2, KI 5, KI 6, KI 10].

“*Initially*, *the Global Fund focused on HIV/AIDS prevention … That was the entry point*. *… Under PHAMIT II*, *one of the hidden goals was to include ART in the health insurance package for migrants to ensure sustainability*. *Second*, *they wanted to make the migrant health insurance scheme accessible by all migrants*, *not only the registered migrants*. *… The movement of PHAMIT was one of the driving factors*, *or the driving force that made a major change to the health insurance policy for migrant workers in Thailand in 2013*. *If you look at the policy changes in 2013*, *the first thing is that ART is covered and the second thing is that it is open to all*, *not only to the registered migrants*.” [KI 6]

This has to be seen against the fact that ART for Thai citizens was only added to the benefit package of the UCS in 2006, with NGOs and their extensive networks of people living with HIV/AIDS, some of which also received funding from GFATM, having been central in advocating the UCS policy change [57].

In addition, other international organizations operating in Thailand provided financial resources for local initiatives in certain areas such as, for example, the Thai-Myanmar border [KI 3, KI 7, KI 11]. Financial support was inter alia given to community-based organizations, which provide micro-health insurance or healthcare for target populations, including migrants.

“*They influence policy by piloting*, *for example*, *new approaches*. *And*, *I think that is exactly what they wanted to do here in case of this health insurance for migrants*. *… They support an alternative concept in order to highlight a gap that exists*, *new approaches that could be relevant for the country*. *So*, *that’s why they support us*. *And*, *they want us to be visible with the results*.” [KI 11]

Local initiatives are aimed at overcoming weaknesses of existing health insurance schemes in terms of e.g. enrolment and premium payment [KI 3, KI 11, KI 17, KI 18]. The SSS has suffered from high leakages as many employers perceive the process as too cumbersome and costly, while migrants consider certain benefits as not attractive, inter alia due to a lack of understanding. The SSS benefits cover sickness, maternity, child allowance, invalidity, death, old-age pension and unemployment and by design focus on long-term employment. The stay of many migrant workers, however, is typically limited to two to four years. Several respondents pointed out that the MoPH has been more flexible and proactive compared to the Social Security Office and other ministries, given that the MHI has been revised to better suit the needs of migrants, by, for example, offering different contract duration options [KI 2, KI 6, KI 14, KI 15]. Key informants further added that migrant workers in the informal sector are, in essence, excluded from voluntarily opting into the SSS, including, for example, migrants working as domestic workers, fishers and agricultural workers with contracts of less than one year [KI 2, KI 14]. It is estimated that about one third of regular migrant workers are working in the informal sector [24].

A positive role was generally attributed to international donors for their ability to give needed impetus and fill important gaps [KI 2, KI 7, KI 8, KI 10, KI 11, KI 18].

“*External actors can provide a small portion of money to lubricate the system*. *There is something that can be done*, *to pilot*, *to move on in a short period of time and to build champions*. *That is something that donors can support with small amounts*.” [KI 2]

Providing financial support has allowed international donors to influence priority setting as, for example, evidenced in the role of the GFATM with respect to the inclusion of ART in the benefit package of the MHI. Moreover, through financial assistance international donors have been in a position to initiate pilots, support evaluations and research and help create an evidence base that influences not only priority setting, but also supports policy formulation and implementation, as well as monitoring and evaluation [KI 2, KI 7, KI 8]. Funding research has enabled international organizations to decide which research to fund and which evidence to use to inform policy-makers and to show what can be done [KI 2, KI 10].

### 3.2 Technical expertise

While the technical expertise of external actors was acknowledged, key informants emphasized that the relevant knowledge and research capacity, especially in the area of health policy and health systems strengthening, is quite strong in Thailand. The role of external actors was felt more pronounced in terms of helping to identify problems and finding solutions, thereby influencing priority setting, policy formulation and implementation to fill remaining gaps.

External actors provided needed feedback and raised important issues and gaps during meetings [KI 1, KI 7, KI 8, KI 9]. By highlighting key problems through their research, external actors also affected the monitoring and evaluation stage. Issues and solutions were framed and promoted by external actors to secure interest and attention.

“*We are taking some sort of steps like “this is your last mile”*. *Migrants are your last mile*. *… You are almost perfect*. *You started your UHC [universal health coverage] when your country had the economic crisis*. *You ran your boat throughout the storm and you are still rolling your boat in a very good shape and you are also teaching or sharing experiences with other countries*, *coaching other countries to do the same*. *… If you do something for migrants*, *you can claim UHC for all*.” [KI 2]

Frames had to be negotiated, agreed upon and coordinated by external actors and their networks. Meetings, seminars and conferences were regularly convened by some international organizations to debate, share experiences and lessons learned several key non-government informants pointed out [KI 2, KI 3, KI 5, KI 6, KI 10, KI 14, KI 17]. Thereby, they created coordination platforms, which was particularly important in an environment where the work of domestic actors suffered from fragmentation and involved partners from several sectors. The ability of international organizations to link stakeholders and create platforms for dialogue at all stages of the policy process was considered their key strength as emphasized by three key informants from international organizations [KI 2, KI 3, KI 14].

“*The problem is how to move it forward*, *make it happen*. *This is one of the challenges*. *There is no central coordination office for migrant health in Thailand*. *There are so many players and there is no focal point*.” [KI 3]

Another key informant, however, emphasized that while stakeholder coordination is important, it was not a key driver of change [KI 4]. Similarly, international organizations were recognized for setting global standards, policies and directions for local players to use as benchmark and reference, which supported the domestic policy process, but these were not considered transformative.

### 3.3 Inter-sectoral leverage

Key informants unanimously agreed that inter-sectoral leverage was exercised through the US TIP Reports, given that Thailand was placed on the Tier 2 Watchlist from 2010 to 2013 and from 2016 to 2017. In 2014 and 2015, Thailand slipped to Tier 3 [58, 59]. In addition, in 2015, the European Commission gave a notice (yellow card) to Thailand for failing to sufficiently tackle illegal, unreported and unregulated fishing [IUU], which was only lifted in 2019. The TIP report ranking, on the other hand, improved to Tier 2 in 2018 [58]. The TIP Reports and also the yellow-carding decision by the European Commission were perceived by all key informants to have had a strong direct impact on efforts to tackle human trafficking and the substandard working conditions in the Thai fishing sector.

“*Thailand is part of the global community and a bad reputation due to the TIP Reports can definitely affect us as we might have to face trade barriers*, *and for the EU IUU carding*, *it is clear that things might get more difficult for our fishery sector*.” [KI 12]

While the effect of the TIP Reports was considered reputational, the EU carding decision was viewed as having had a direct impact on trade and investment flows. The impact on tourism, however, was considered rather small by all key informants except one, who pointed out that some hotels started to promote fair working conditions for migrant workers to alleviate any impact and ensure that their contracts with tour operators abroad are not terminated [KI 17].

In 2013, apart from the inclusion of ART in the benefit package of the MHI, another two major changes took place, (i) a health insurance card for migrant children below age seven was launched, and (ii) migrants (aliens who are not covered by the SSS; as opposed to migrant workers) could purchase the MHI for an annual premium of THB 2,200 (plus THB 600 for the medical check-up (Table 1) [20, 23]. These changes, especially the health insurance card for migrant children below age seven, were perceived as efforts to improve Thailand’s global image.

“*The health insurance card for migrant children was due to Tier 2 Watchlist*. *Trying to produce the image that we are protecting people*, *especially the vulnerable groups*. *… The insurance premium is only THB 1 per day*. *The premium is a gimmick*, *there is no evidence*. *This is a truly political judgement*. *… The change from migrant worker to migrant is also linked with the Tier 2 Watchlist*. *We are trying to cover everyone*.” [KI 4]

“*The Yingluck government wanted to demonstrate at the UN meeting that it is committed to providing everybody in Thailand with greater access to healthcare*, *most notably migrant women and children*.*”* [KI 15] (Yingluck Shinawatra and the Pheu Thai Party won the 2011 elections, but efforts to remove her government from office started immediately after the elections [26].)

“*At that stage*, *it could have been a politically correct scheme to open up for Thailand*, *knowing that many kids are left behind*. … O*pening it up to healthy people is the most easily given right*. *It is also a politically viable option*.” [KI 18]

The 2013 changes, like prior changes, however, occurred following cabinet resolutions, given that the MHI lacks a sound institutional set-up. Key informants voiced strong concerns about the dependence on cabinet resolutions and the uncertainty surrounding these, repeatedly pointing out that the MHI is not based on a legislative act like for example the SSS, which was established under the 1990 Social Security Act [KI 3, KI 8, KI 11, KI 14, KI 15, KI 16].

The US TIP Reports and the EU-IUU carding decision also helped to increase awareness among civil society and triggered a crucial domestic force for change as investigations, which were widely publicized throughout the media, showed that government officers were involved in human trafficking crimes [KI 1, KI 2, KI 6, KI 7]. The publicly unfolding revelations could not be accepted [KI 1, KI 14].

“*There were big scandals everywhere related to human rights abuses that cannot be tolerated*. *The media around the world followed*. *It was a global issue at that time*. *When the media found out about the involvement of government officials it was a really big issue*. *… Thai people could not accept this*. *The government had to solve this issue*.” [KI 7]

“*We have the term in Thai “อารยะประเทศ” [pronounced*: *aa-ra-ya pra-thet; civilized country]*. *We want to be an “อารยะประเทศ”*, *a civilized nation*, *and civilized nations have standards*. *… The majority of Thai people believe in modernisation and Japan and the West became the benchmark*. *When you have people saying things against you publicly*, *you become very sensitive because you want to be respected*. *This is our culture*.” [KI 1]

Criticisms of Thailand’s efforts in fighting human trafficking, especially through international media coverage, were perceived by all key informants to have affected the country’s international standing and reputation and thereby influenced priority setting, which indirectly affected the MHI.

The 2014 coup d'état in Thailand and resulting changes in political authority were identified by key informants as amplifiers for the effect of inter-sectoral leverage. The NCPO announced that law enforcements against irregular migrants would be strengthened, which inter alia resulted in a mass exodus of Cambodian migrants [23, 60].

“*The military-led government is not affected by political influence and so they have firm control over the policy*: *when they say “no more”*, *they mean it*. *They will also work to push illegal migrants remaining in Thailand back to their countries*. *It is clear that if migrants would like to work in Thailand*, *the only way they can come to our country is through the process under an MoU*. *They need to register*. *… Given the pressures from the EU IUU carding and TIP Reports*, *the new labour laws set harsh punishments for violations*, *for both employers and employees*. *This government takes this issue seriously and as a priority*.” [KI 13]

The government’s zero-tolerance approach towards illegal migration was considered a response to the TIP Reports and an attempt to improve the military’s global reputation following the coup d'état [KI 8, KI 14, KI 15, KI 17]. Yet, as key industries are dependent on unskilled workers from the three neighbouring countries, several measures were subsequently implemented to stem the outflow of migrant workers, including temporary amnesty programmes [KI 4, KI 6, KI 18].

In 2014, OSSC were established in all provinces under a multi-sectoral approach to facilitate the registration of undocumented migrant workers and their dependants during announced periods of time, which indirectly influenced the MHI by making it part of the OSSC documentation system. To improve affordability, the price for the medical check-up and the annual MHI premium was reduced (Table 1). At the same time, many hospitals stopped selling the MHI to unregistered migrants given legal uncertainties [KI 5, KI 10, KI 14, KI 15, KI 16].

Although the OSSC were re-opened from 2017 to 2018, further amnesty periods will not be available from 2018 onwards, given recent revisions of legislation and policies [KI 10, KI 12, KI 13]. The 2017 Royal Ordinance on the Management of Foreign Workers Employment B.E. 2560 and its 2018 amendment, for example, aim at improving the protection of migrant workers’ rights in Thailand. About 1.2 million migrants registered with the OSSC between July 2017 and June 2018 alone [16]. The number of migrant workers enrolled in the SSS increased from 357,643 in September 2013 to 1,107,426 in September 2018 [24].

## 4. Discussion

Providing grants to strengthen services for key populations, funding pilot projects and financially supporting research enabled international donors to exert some influence on all stages of the policy process underpinning Thailand’s MHI, which is in line with the literature that focuses on aid-dependent countries [1, 3, 4]. External actors were particularly influential at the agenda-setting stage though by not only financing innovative approaches, but also advocacy to promote these. The ability of external actors to influence the agenda aligns with the second face of power, that is power through agenda-setting. Financial power is derived from the conditional provision of financial resources and financial resources are an important source of power [9, 30, 31, 33]. Although the financial power of many international donors has declined, given more intense competition for funding, the findings show that the provision of small amounts still allows external actors to exercise influence.

Epistemic power, on the other hand, stems from technical expertise, i.e. the ascribed advantage external actors have in generating and using knowledge and information [3, 13]. Scientific knowledge and skills of external actors, however, are on par with domestic expertise according to key informants and, hence, were considered not important in case of Thailand’s MHI, which is contrary to the findings in the literature [3, 8]. Some key informants, however, pointed out that external actors played a meaningful role in terms of bringing stakeholders together at all stages of the policy process as they have strong coordination mechanisms and convening power. This positive view, held by key non-government informants though, is contrary to Biesma et al. [61] and Hanefeld [2], who found that donors distort domestic coordination efforts. In addition, coordination and collaboration among donors in aid-dependent countries can increase the bargaining power of external actors vis-à-vis domestic actors [3]. In this study, however, coordination platforms were recognized for bringing external and domestic actors together and to facilitate the exchange of experiences and allow a discussion of current issues. The ability to draw on and create epistemic communities has traditionally been a key channel for international donors to exercise power [62]. Yet, claims to moral authority and expertise that allow agenda-setting and preference-shaping (the second and third faces of power) have increasingly been challenged by the emergence of new actors and weakened by the decline in financial resources [62]. While technical expertise in the form of scientific knowledge and skills was not found important in case of Thailand’s MHI, coordination mechanisms and framing strategy mattered. The findings show that coordination mechanisms were perceived by some key informants to have helped overcome the challenges posed at all stages of the policy process by the fragmentation of domestic actors. The MHI was framed by external actors as a last-mile issue to ensure that no one is left behind, in line with the norms of the sustainable development goals, which allowed external actors to gain attention, especially in light of Thailand’s standing in the global health arena. In addition, tractability may have also played a role. The solution to the last-mile problem is relatively straightforward, namely coverage expansion. Technical expertise may, therefore, have been less important than in other contexts. These observations resonate well with the literature. The importance of framing, frame alignment with norms, as well as tractability for agenda-setting is highlighted in several studies [36, 38–44].

In addition, the finding that the provision of financial resources and technical expertise were perceived to have influenced all stages of the policy process underpinning the MHI, contradicts the findings in Khan et al. [3], who showed that the influence of external donors through these two channels differed depending on the stage of the policy process. Analogous Khan et al. [3] and despite contextual differences, the analysis of inter-sectoral leverage showed that this channel mainly influenced priority setting. The analysis by Khan et al. [3] revealed that power to influence priority-setting is exercised through the ability of external actors in health to affect a country’s global standing in non-health sectors. In this study, however, external actors pushed for changes in a non-health sector, which created spill-over effects on the migrant health insurance.

Placing Thailand on the watchlist of the US TIP Report had serious reputational effects, while the European Commission’s yellow card to Thailand for failing to sufficiently tackle IUU threatened the fisheries sector. TIP reports and EU carding system use the instruments of naming, shaming and sanctioning sequentially to pressure countries to change their behaviour. The TIP reports place countries into one of four tiers based on their anti-TIP efforts, with Tier 1 being the best and Tier 3 the worst tier [51]. Under the EU carding system, countries that fail to follow set standards can be pre-identified (yellow-carded) and, if necessary, identified (red-carded) by the European Commission if they do not put in place required reforms [63]. Crucially, the rankings and ratings enable issuing countries to enter a dialogue and outline actions to improve the score in the future. While the TIP reports are a diplomatic tool linked to foreign aid sanctions, the EU carding system can ultimately lead to trade bans. Mundy [63], for example, reported fluctuations in import flows and declines in seafood imports to the EU, following the EU IUU regulation’s entry into force in 2010 and subsequent carding decisions. Thailand is one of the world’s largest exporters of shrimp, canned tuna, squid and cuttlefish and the EU is Thailand’s third largest trading partner overall. A red-carding decision would have resulted in a ban on the country’s seafood exports by EU members [63]. The sequential approach of the TIP reports and the EU carding system, hence, entails a transition from softer to harder power, and aligns with all three faces of power.

The TIP Reports and the carding decision, coupled with widespread international media coverage, created pressures and forced the Thai government to tackle problems surrounding unregistered migrants and to improve the country’s international reputation. These efforts implicitly affected migrant health and the migrant health insurance. The international shaping of norms was identified as an important driver of agenda setting in several studies [34, 36, 42]. Sridhar and Gómez [11] in their study on Brazil, India and Russia further showed that negative media coverage exercises soft pressure by tarnishing a country’s international standing and influence. Revelations of government officials’ involvement in human rights violations were focusing events that highlighted the severity of the problem and triggered strong domestic advocacy. Focusing events have agenda-setting power as for example discussed in Shiffman [42]. Key informants emphasized that after the 2014 coup, reputational effects were very strong and, coupled with the threat of trade sanctions, led to improvements.

Inter-sector leverage was thus found to be intertwined with the power of holding political authority, a relationship that was not identified a priori. The international condemnation of the 2014 coup [64, 65] accelerated the effect of the TIP Reports and the EU carding decision and further damaged Thailand’s international reputation according to key informants. Besides, as the findings in Thyne et al. [66] demonstrate, domestic and international reactions in protest of a coup have regime-shortening effect, which may have put additional pressure on the military-led government to improve its reputation. As the NCPO possessed political authority to advance migrant-related policies and apply pressure on key domestic actors, including health policy actors, several changes to migrant-related policies took place post-2014. In recognition of Thailand’s progress in dealing with the shortcomings in the fisheries legal and administrative systems, the yellow card was lifted in January 2019, about two months prior to the first post-coup election [58].

These findings show that domestic and foreign policy interests, most notably the desire to protect the country’s reputation and trade, motivated action on IUU to comply with international norms, which also affected the MHI. How countries are viewed by their citizens and the world is more important than ever in today’s globalized world with increasing data and information flows. Countries have to manage their reputation and promote their attractiveness to be able to compete in areas such as trade, investment and tourism, and democracy and human rights, for example, are sources of attraction as pointed out by Nye [67].

The interconnectedness of health and foreign policy and other policy domains, as well as the complexity of these nexus, has long been recognized [68–73] and the findings of this study add to the discussion. While foreign policy interests, especially national security and economic interests, often overshadow health objectives [68, 70], these “*selfserving motives for state action on health do not have to lead to poor outcomes*” as stated in Feldbaum, Lee and Michaud [68] and illustrated by the case of Thailand’s MHI.

Thailand may perhaps be an exceptional country though, given its strong domestic health capacity, supporting mechanisms and prominent role in the global health arena. It is also important to bear in mind that this study narrowly examines the role of external actors in shaping the MHI, and neither discusses the crucial role of domestic governmental and civic actors nor their interaction over time in depth. Yet, as mentioned, most pressure ultimately stemmed from domestic stakeholders at the national and local levels, especially until 2013. While this study focused on the influence of external actors on the MHI, Thailand’s impressive progress with achieving universal health coverage may have allowed it to also exert influence on external actors, who have been under increasing pressure to show results given the general debate on aid effectiveness. In other words, the role of external actors may have increasingly become endogenous to the (aid-independent) recipient country, highlighting the importance of contextual factors. Future research should focus on the endogeneity of external actors’ influence in Thailand and other aid-independent countries.

## 5. Conclusion

Existing literature typically focuses on the influence of international donors, especially through global health initiatives such as the GFATM, on country health priorities and policies [1–7]. Focusing on a specific health policy in an aid-independent country, this paper examined the influence of external actors through various channels on the stages of the policy process. The results show that external actors played a different role in aid-independent countries, which is important to understand as more and more countries are graduating to (upper) middle-income status.

Providing some financial support, even if small overall, allowed external actors to exert influence and help identify and fill important gaps. In contrast to the literature, the key aspect of technical expertise was perceived to be the ability of international organizations to link the various actors, given the fragmentation of Thailand’s health insurance system. Inter-sectoral leverage, by tarnishing the country’s reputation was found very important, if not most important, in terms of indirectly affecting migrant health insurance through efforts aimed at dealing with problems of human trafficking, especially after the 2014 coup d'état, which had an accelerating effect. This study helps understand the changed role external actors can play in filling health system gaps in aid-independent countries.

## Supporting information

S1 File Interview guide. (DOCX)
