# Supplementary material for: The role of external actors in shaping migrant health insurance in Thailand
Source: PLoS One. 2020 Jul 2;15(7):e0234642. doi: 10.1371/journal.pone.0234642 (PMC7332068; doi:10.1371/journal.pone.0234642)
Supplement: S1 File — (DOCX) [file pone.0234642.s001.docx]

Interview guide

**Questions about respondent’s role**

1. How does your work relate to health insurance for migrant workers in Thailand?
2. Do you think your work informs/influences policy-making on health insurance for migrant workers in Thailand? In what ways?

**Questions about health insurance for migrant workers in Thailand**

1. Briefly explain the current health insurance coverage for documented and undocumented migrant workers in Thailand and its evolution.

- Social Security Scheme
- Compulsory Migrant Health Insurance
- Others (such as private insurance)

(*Focus on Social Security Scheme versus Compulsory Migrant Health Insurance and complementarities.*)

1. How is the Compulsory Migrant Health Insurance monitored and evaluated? What organization bears the primary responsibility for M&E? What framework is used? How are the findings used and disseminated?
2. What do you perceive to be the Compulsory Migrant Health Insurance’s key weakness? Why?
3. In which institution do you think is the overall responsibility for migrant worker health insurance primarily vested?

**Questions about external influences on health insurance for migrant workers in Thailand in general and Thailand’s Compulsory Migrant Health Insurance in particular**

1. From your point of view, are there any external actors who influence policies related to health insurance for migrant workers in Thailand?
   1. If you do not think that there are any external actors who influence policies related to health insurance for migrant workers in Thailand, please elaborate and explain why. [*Interviewer asks probing questions, especially about key domestic actors who influence policies related to health insurance for migrant workers*]
   2. If you do think that there are external actors that influence policies related to health insurance for migrant workers in Thailand, please proceed.
2. Who are these external actors and what is their role? Who do you perceive to be the main external actors that influence policies related to the Compulsory Migrant Health Insurance? Why?
3. Through which channels do external actors exert influence (financial resources, technical expertise, intersectoral leverage (e.g. travel and trade restrictions), others)? In your opinion, which of these channels is the strongest? Why?
4. Do you perceive external influences to be appropriate and constructive? Why or why not?
5. Do you think external actors are better able to produce, interpret and disseminate knowledge? Why or why not? Does this ability give them an advantage and allow them to exert influence?
6. Focusing on the Compulsory Migrant Health Insurance, please explain the influence of external actors on the various stages of the policy process, i.e. priority setting, policy formulation, policy implementation, monitoring and evaluation.
7. In your opinion, does the channel of inﬂuence differ depending on the stage of the policy process? In what way? Please explain.
8. Is there anything else we have not discussed that you would like to share? Do you have any questions?

Thank you for your time!

Notes:

1. Given the controversial use of the terms used to refer to “migrants” in the Thai language and in policy documents [20, 22, 23, 25], the term “migrant worker” was chosen for the interview guide to emphasize that the focus of this study is on migrants from neighbouring countries as opposed to other categories of migrants.
2. The migrant health insurance of the Ministry of Public Health (MHI) is also referred to as the “Compulsory Migrant Health Insurance (CMHI)” [19], the “Health Insurance Card Scheme (HICS)” [22, 23, 25] or the “Migrant Health Insurance Scheme (MHIS)” [20]. While CMHI was used in the interview guide, MHI was eventually used in the research article to avoid ambiguity.
